# Annexin A1 and A2 in inflammatory bowel disease pathogenesis: exploring new avenues for diagnosis and treatment

**DOI:** 10.3389/fimmu.2025.1725965

**Published:** 2026-01-02

**Authors:** Najib Muaamer Faed Murshed, Praveenkumar Shetty, Pavan K. Jayaswamy, Ankeeta Menona Jacob, Samah Saleh Ahmed Alawadhi, Kishan Prasad Hosapatna Laxminarayana

**Affiliations:** Department of Pathology, KS Hegde Medical Academy, Nitte (Deemed to be University), Mangalore, India

**Keywords:** inflammatory bowel disease, annexin A1, annexin A2, biomarker, pathogenesis, Ulcerative Colitis, Crohn’s Disease

## Abstract

Inflammatory bowel disease (IBD), which includes Crohn's disease and ulcerative colitis, is a significant global health burden with gastrointestinal inflammation resulting from dysfunctional immune responses and chronic inflammation. This review synthesizes the roles of annexin A1 (AnxA1) and annexin A2 (AnxA2) in the pathophysiology of IBD, their diagnostic biomarkers, and therapeutic potential. AnxA1 interacts with FPR2/ALX receptors, inhibiting the release of pro-inflammatory cytokines and promoting epithelial cell repair. AnxA2 exhibits dual roles by interacting with S100A10. AnxA2 can induce NF-κB activation, promoting pro-inflammatory cytokine release and plasminogen activation. On the other hand, AnxA2 activates the TRAM-TRIF pathway, inhibiting NF-κB activation, promoting production of anti-inflammatory cytokines, fibrinolysis, and restoring tight junctions. Their modulation of NF-κB pathways shapes the molecular landscape of IBD. AnxA1 and AnxA2 are non-invasive plasma biomarkers that improve subtype-specific diagnostic accuracy compared to C-reactive protein. In the therapeutic context, AnxA1 mimetics and AnxA2 inhibitors reduce inflammation and promote healing, potentially in conjunction with anti-TNF drugs or nanoparticle delivery. Longitudinal studies and clinical trials are essential to identify the gaps in the standardization of testing and cytokine network interactions. AnxA1 and AnxA2 have the potential to transform the development of precise diagnostics and personalized therapies, redefining the management of IBD.

## 1 Introduction

**Figure 1:**
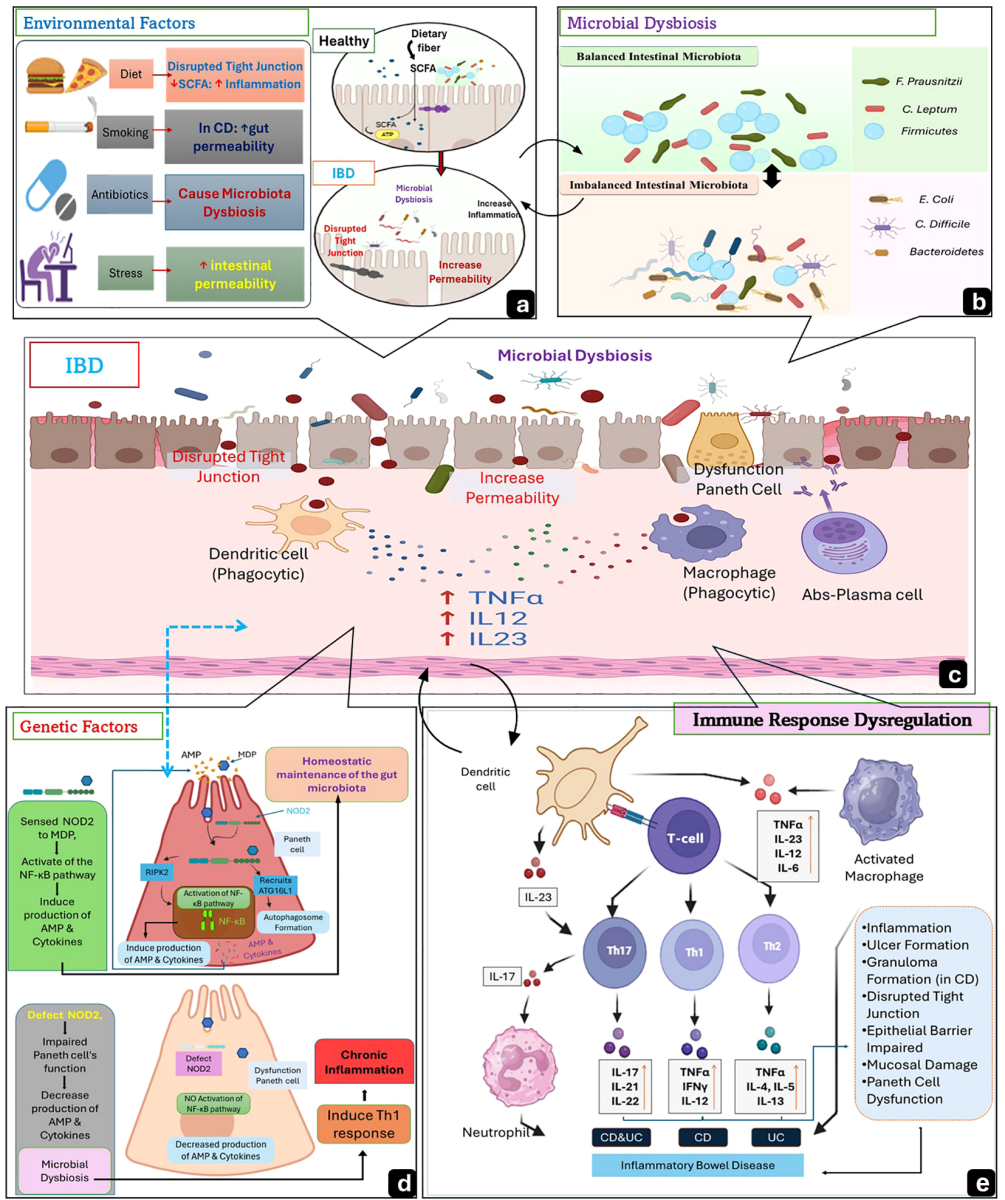
Schematic illustration of the Factors of Etiopathogenesis of IBD. **(a)** Environmental factors, such as a high-fat, high-sugar diet, disrupt tight junctions, while a low-fiber diet reduces short-chain fatty acids (SCFAs), thereby impairing epithelial barrier integrity. Smoking (mainly in CD) increases gut permeability, antibiotics eliminate beneficial bacteria, leading to dysbiosis, and stress disrupts the gut-brain axis, promoting inflammation. **(b)** Microbial dysbiosis occurs when there is an imbalance in gut microbiota, with a decrease in beneficial bacteria and an increase in pathogenic microbes. **(c)** Inflamed intestinal mucosa in IBD shows mucus depletion due to Goblet cell dysfunction, disrupted tight junctions, increased permeability, and microbial translocation into the lamina propria. **(d)** Genetic factors such as NOD2 mutations impair Paneth cell function, reducing antimicrobial peptide (AMP) production and contributing to dysbiosis. **(e)** Immune response dysregulation occurs when CD4+ T cells are activated and then differentiate into Th1 (mainly in CD)cell, Th17 (commonly in CD), and Th2 (mainly in UC), releasing pro-inflammatory cytokines that drive chronic inflammation. Created by the author using BioRender and Microsoft PowerPoint.

Inflammatory Bowel Disease (IBD) is a group of chronic inflammatory conditions involving the gastrointestinal tract (GIT), especially the lamina propria, characterized by relapses and remission. That has a substantial impact on the health, quality of life, and psychological dimensions of IBD patients and is subclassified into ulcerative colitis (UC) and Crohn’s disease (CD) (1, 2). The global prevalence and burden of IBD are rising, with significant regional and national differences in prevalence and trends. As per 2017 data, there were 6.8 million cases of IBD worldwide. The age-standardized prevalence rate increased from 79.5 per 100,000 people in 1990 to 84.3 per 100,000 in 2017 (3). Incidence rates of IBD show significant variation by region. Europe saw rates ranging from 0.97 to 57.9 cases, North America ranged from 8.8 to 23.14, and Asia and the Middle East ranged from 0.15 to 6.5 (4). In India, the incidence and prevalence of IBD are higher than previously believed, with an incidence of 9.3 per 100,000 and nearly 1.5 million cases (5, 6). Due to convergent clinical and pathological characteristics, it can be challenging to distinguish between different IBD subtypes of UC and CD (7, 8). These subtypes of IBD share common symptoms such as abdominal pain, recurrent diarrhea, and rectal bleeding (which occurs more frequently with UC than with CD) (3, 9). Patients with IBD experience a wide range of consequences throughout their condition, such as intestinal bleeding, toxic megacolon, formation of abscesses and constrictions, and fistulizing conditions (4, 5). UC is characterized by persistent inflammation that damages the colon's mucous membrane, which starts at the rectum and frequently causes erosions and ulcers. The CD is featured by patchy, granulomatous inflammation that can affect any part of the digestive tract and is not continuous, often leading to the formation of fistulas (7, 10). The etiology of IBD is not well defined. The etiopathogenesis mechanisms in IBD involve environmental influences, genetic susceptibility, dysregulated immune responses, and microbial dysbiosis, as shown in **Figure 1**. These factors lead to intestinal barrier dysfunction, resulting in bowel inflammation (11). The diagnosis of IBD is made by physical examination and the patient's history in combination with colonoscopy, histological and radiographic tests, and non-specific tests for biomarkers from blood and/or stools (12). Inflammation in IBD is marked by imbalanced levels of specific molecules, many of which are validated but not routinely tested. These include molecules related to the inflammatory acute-phase response, coagulation and fibrinolysis, proteinase inhibitors, transport proteins, other serum proteins, and cytokines (13).

Dysregulation of immune response characterizes the Pathogenesis of IBS, which compromises the intestinal epithelial barrier integrity, leading to chronic inflammation, by hypersecretion of pro-inflammatory mediators involving Tumor Necrosis factor-alpha(TNF-α) and Interleukin-6 (IL-6), which release by overactive T-helper-1 (Th1) and T-helper-17 (Th17) cells in CD or T-helper-2(Th2) cell in UC (14). These cytokines induce nuclear factor-kappa B (NF-κB) activation, which amplifies the inflammation cascade initiated by immune dysregulation (15). Therefore, in countering the inflammation caused by these pro-inflammatory cytokines, endogenous pro-resolving proteins such as Annexins play critical roles in maintaining epithelial barrier integrity, stabilizing tight junctions, and mitigating barrier disruption (16). Annexin A1 (AnxA1) is a glucocorticoid-regulated protein that promotes inflammation resolution through multiple mechanisms, including inhibition of NF-κB signaling, enhancement of IL-10 production, limitation of neutrophil recruitment, and promotion of macrophage-mediated efferocytosis; these actions support mucosal healing in preclinical colitis models and are associated with mucosal recovery in human samples (9, 17, 18). Annexin A2 (AnxA2) contributes to membrane–cytoskeleton dynamics (exocytosis, endocytosis, phagocytosis), cell migration, and plasminogen activation, and has context-dependent functions in inflammation and tissue repair; in colitic settings, AnxA2 can amplify NF-κB–driven cytokine production and epithelial responses, while in other contexts it supports fibrinolysis and wound closure (19, 20,).

To control the disease, IBD frequently requires long-term treatment based on a mix of medications. The range of IBD treatment choices has expanded significantly over the last several years. Medications such as aminosalicylates, Corticosteroids, immunomodulators, and biologics are combined with other general interventions and, in some instances, surgical resection to control symptoms (12). It has been demonstrated that biological therapy, such as Adalimumab, Infliximab, and Certolizumab, improves the quality of life for patients with IBD by blocking the pro-inflammatory cytokine tumor necrosis factor-alpha (TNF-α). However, some individuals do not react to biological therapy for various reasons, including the immune system state. This variability makes patient monitoring, including measuring medication levels in plasma, essential during treatment (21). When treating IBD in its acute stages, Glucocorticoids (GCs) have been recommended to induce the disease into remission. GCs exhibit significant anti-inflammatory effects by suppressing the migration and proliferation of practically all immune cells, depending on the disease's severity (22). It has been demonstrated that several pro-resolving mediators can control critical phases of inflammation to induce resolution, specialized pro-resolving lipid mediators (SPMs), and proteins such as annexins (23).

Recent single-cell and multi-omics studies refine the cellular map of annexin expression in human IBD: AnxA1 signals are detected in epithelial cells, stromal compartments, and certain T-cell subsets, while AnxA2 transcripts are enriched in macrophage populations in inflamed colonic mucosa (24–26). These datasets indicate cell-type and treatment-context dependence of annexin expression, and they motivate cautious evaluation of annexins as candidate biomarkers or therapeutic targets. Inter-study heterogeneity, driven by patient age, microbiome composition, medication exposure, sample type (biopsy versus blood), and analytic platform (bulk versus single-cell versus proteomics), limits the generalizability of individual reports and underlines the need for standardized assays and larger, longitudinal cohorts (27, 28).

### 1.1 Literature search strategy

A targeted literature search was conducted in PubMed, Scopus, and Web of Science for all available years up to 2025, using Annexin-related terms (“Annexin A1”, “AnxA1”, “Annexin A2”, “AnxA2”) combined with inflammatory bowel disease terms (“inflammatory bowel disease”, “ulcerative colitis”, “Crohn* disease”, “colitis”). The search retrieved 186 records, comprising 55 PubMed (AnxA1+IBD = 52; AnxA2+IBD = 3), 68 Scopus (AnxA1+IBD = 61; AnxA2+IBD = 7), and 63 Web of Science (AnxA1+IBD = 61; AnxA2+IBD = 2) records. After removing 101 duplicates, 85 unique studies remained. As this is a narrative (non-systematic) review, broad inclusion criteria were applied, permitting original studies from human IBD cohorts, validated animal colitis models, and *in vitro* mechanistic systems examining annexin signaling, mucosal immunity, epithelial biology, or biomarker development. Review articles and mechanistic overviews were consulted when they contributed relevant conceptual or translational context. Non–peer–reviewed materials, conference abstracts, and articles lacking extractable biological relevance were excluded.

**Figure 2:**
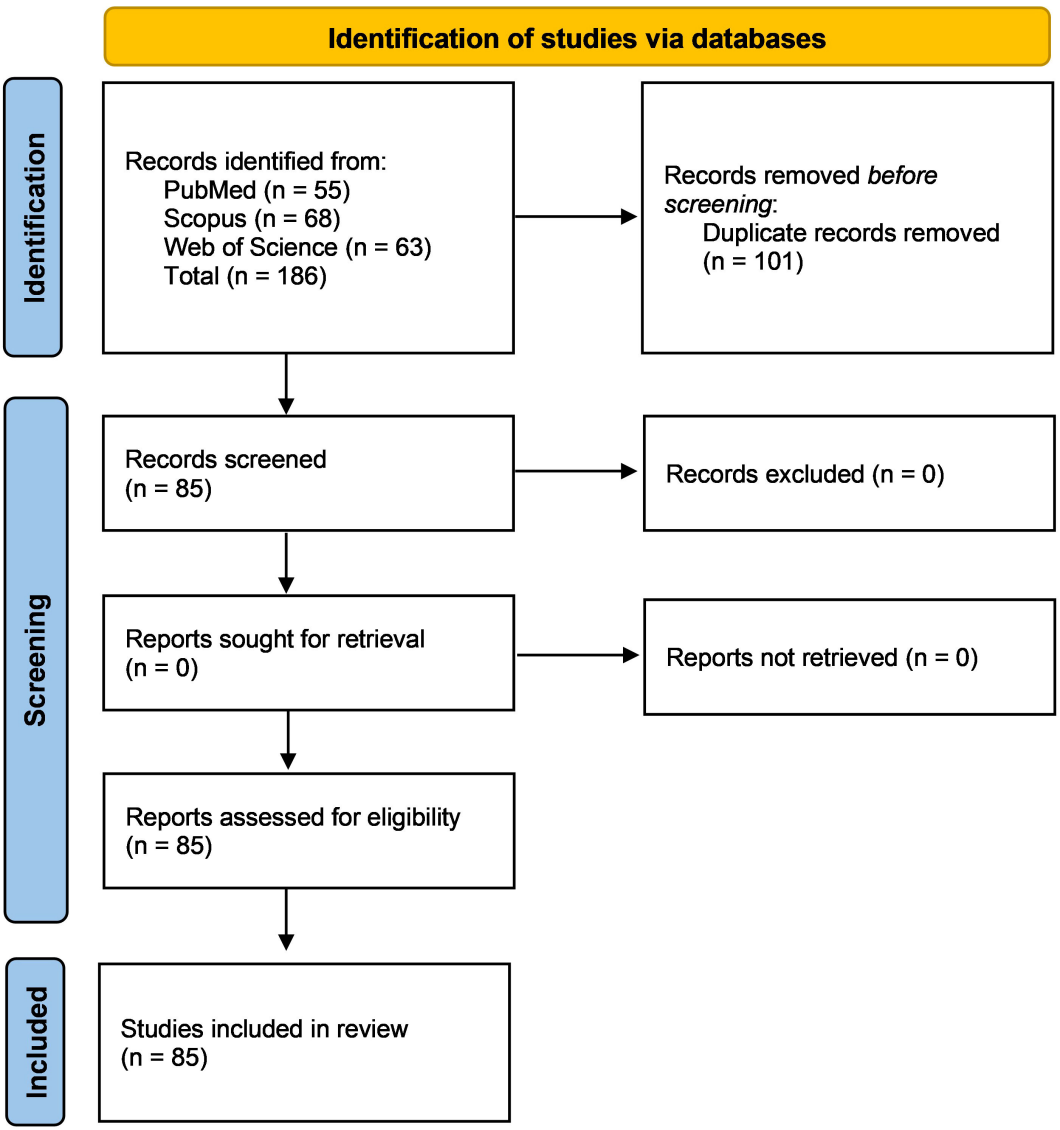
PRISMA-style flow diagram.

For clarity, evidence was organized into three categories: human clinical evidence (biopsy analyses, circulating biomarkers, proteomics, and single-cell datasets), animal evidence (e.g., DSS, TNBS, IL-10–deficient, and other validated colitis models), and *in vitro* evidence (immune cells, epithelial cells, and intestinal organoids). This framework allows distinction between preclinical mechanistic insights and emerging clinical observations, supporting a coherent and translational interpretation of Annexin biology in IBD. A simplified PRISMA-style flow diagram summarizing the search and screening steps is presented in **Figure 2**.

## 2 Etiopathogenesis of IBD

### 2.1 Etiology of IBD

Inflammatory bowel disease develops from a multifactorial interplay involving environmental exposures, microbial dysbiosis, genetic susceptibility, and dysregulated immune responses. Environmental factors, including smoking, diet, medications, antibiotics, and psychosocial stress, influence intestinal permeability, microbiome composition, and mucosal immune function (11 ).Recent multi-omics studies further confirm that environmental stressors reshape gut microbial metabolites and epithelial–immune communication, influencing disease onset and relapse (29).

#### 2.1.1 Environmental factors

Environmental factors strongly influence the etiopathogenesis of IBD (11), where smoking, food style, medications, stress, and psychological components are important factors (30, 31). Smoking has a contradictory impact on different types of IBD. In CD conditions, smoking is harmful, raising the risk of disease onset and postoperative complications. Smoking does not improve UC disease progression (32, 33). Diet plays a crucial role in IBD (34). High consumption of total fats, polyunsaturated fatty acids, omega-6 fatty acids, and meat is associated with an increased risk of CD and UC. In contrast, diets rich in fruit, fiber, and vegetables are linked to a reduced risk of both diseases (35). High doses and frequent use of nonsteroidal anti-inflammatory drugs are associated with a higher risk of CD and UC, whereas aspirin has not shown a significant connection (36). Antibiotic intake, mainly in early life, is significantly associated with increased IBD risk due to its effect on the developing intestinal microbiome (37). Other medications, like anti-contraceptives and statins, also raise the IBD risk (38). Psychological stress, including anxiety and depression, can exacerbate IBD by disrupting immune function through mechanisms such as the hypothalamic-pituitary-adrenal axis, bacterial-mucosal interactions, and neuroimmune mucosal mast cell circuits (39). Integrative analyses show that chronic stress reshapes Th17-related cytokines and microbial metabolites, creating a relapse-prone inflammatory state (25).

#### 2.1.2 Microbial imbalances factors

The intestinal microbiome plays a crucial role in human health as a key link between the external environment and the intestinal mucosa (40). Microbial dysbiosis, characterized by a decrease in microbiota diversity, significantly impacts health (41). The gut microbiota is essential for balancing T helper (Th) cells and T regulator (Treg) cells, eliciting the body's inflammatory response. When the composition or function of the microbiota is disrupted, it disrupts the mechanism, leading to intestinal inflammation (40). In patients with IBD, there is a significant disruption in gut microbiota, characterized by a decrease in beneficial, anti-inflammatory bacteria like *Faecalibacterium prausnitzii* and *Clostridium leptum*, and an increase in harmful, pro-inflammatory bacteria such as *Escherichia coli* and *Clostridium difficile* (42, 43). This imbalance includes reducing *Firmicutes* and increasing *Proteobacteria* and *Bacteroidetes (*44*).* The reduced short-chain fatty acid(SCFA)-producing bacteria impact Treg cell differentiation and epithelial growth, contributing to intestinal inflammation (45). Additionally, the gut microbiota in IBD patients exhibits reduced diversity and stability compared to that of healthy individuals, which further exacerbates disease progression and alters the immune environment of the gut (46).Recent spatial multi-omics analyses highlight microbial–immune clustering in inflamed UC mucosa, where certain bacterial taxa colocalize with activated myeloid cells and IL–23–rich niches (47).

#### 2.1.3 Genetic factors

Genetic susceptibility plays a crucial role in our understanding of IBD, particularly CD, compared to UC (48). Higher concordance rates in monozygotic twins and a significantly increased risk for first-degree relatives (49). Rare genetic variants, such as mutations in the XIAP and IL10RA genes, have been associated with severe early-onset IBD (50). Genome-wide association studies have identified 201 genetic loci linked to CD and UC, suggesting shared inflammatory pathways (51). Notable genes include NOD2, ATG16L1, IRGM, and IL23R. However, these loci account for only 20%-25% of the disease's heritability, highlighting a *genetic vacuum (*52*).* Current long-read transcriptomic research is increasingly focusing on noncoding RNA dysregulation and altered polyadenylation profiles in UC mucosa, indicating that the regulatory RNA architecture contributes substantially to the disease phenotype (53). Epigenetic mechanisms, including DNA methylation and microbiome-responsive chromatin changes are emerging contributors to the unexplained (heritability gap) (31, 54).

#### 2.1.4 Immune dysregulation factors

Both innate and adaptive immune mechanisms contribute significantly to IBD pathogenesis (11). A Th1-mediated immune response is the primary driver of CD (55), while an atypical Th2 response is associated with UC (56). Additionally, recent studies have highlighted the crucial role of Th17 cells in the inflammatory processes of IBD, particularly in CD (57). Th1 and Th17 responses are associated with elevated IFN-γ, IL-17A, and IL-12 levels. On the other hand, an atypical Th2 response is characterized by reduced IL-4 levels and increased levels of IL-13 and IL-5 (58). IL-23 is crucial for innate and adaptive immunity, with genetic polymorphisms in IL23R associated with CD and UC. IL-23 significantly contributes to chronic intestinal inflammation by promoting Th17 cytokine production (11). Elevated levels of IL-17A are observed in the mucosa of both CD and UC compared to normal gut tissue (59). In CD, IL-17 and IL-23 play crucial pro-inflammatory roles. IL-23, a member of the IL-12 family, stimulates Th17 differentiation by promoting Th17 gene expression. IL-17 and IL-23 signaling cascades produced various pro-inflammatory substances, including TNF, IFN-γ, and IL-22 (60). During inflammation, intestinal macrophages produce TNF, which is essential for the development of colitis (61). Increased macrophage infiltration in UC produces several inflammatory cytokines, which could affect carcinogenesis (62). Single-cell studies highlighted that the tissue-resident immune subsets, including TRM cells, inflammatory monocytes, and IL-23-responsive macrophages, are central drivers of chronicity (26, 63).

### 2.2 Normal architecture and disruption of the intestinal epithelial barrier

**Figure 3:**
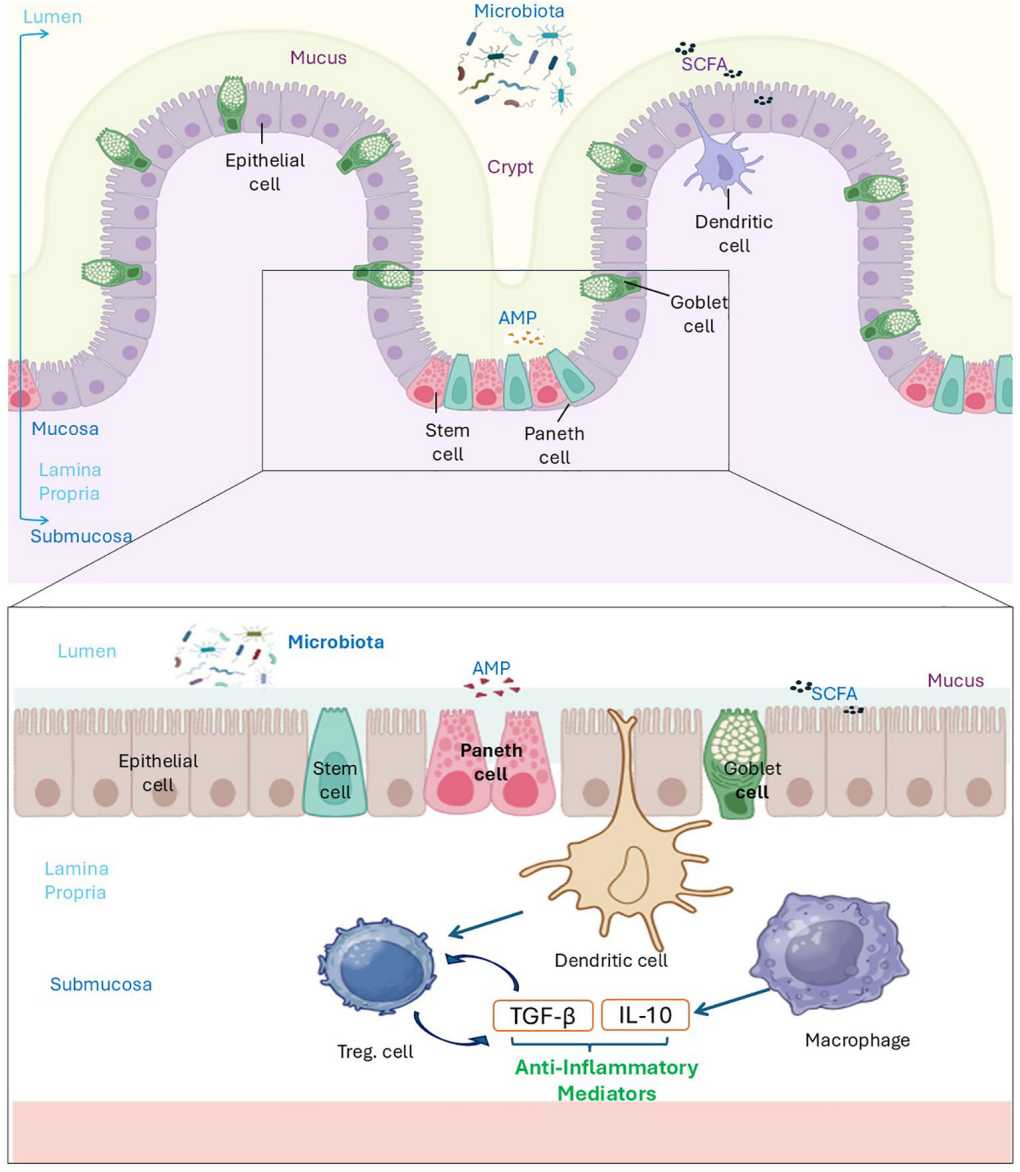
Normal structure of intestinal mucosa in a healthy state. Epithelial cells form the main structural component of the intestinal mucosa, tightly connected by tight junction proteins to regulate permeability. Goblet cells produce mucus, creating a protective layer against microbial invasion. Paneth cells secrete antimicrobial peptides (AMPs) to control microbial populations. Dendritic cells, located between the intestinal epithelium and lamina propria, capture translocated microbiota and produce TGF-β, promoting the generation of Tregs. Additionally, macrophages secrete IL-10, an anti-inflammatory cytokine that helps maintain immune homeostasis. These specialized cells preserve barrier integrity and immune balance in the intestinal mucosa. Created by the author using BioRender and Microsoft PowerPoint.

The intestinal epithelial barrier consists of a single layer of tightly connected intestinal epithelial cells (IECs), forming the frontline between luminal microbes and underlying immune tissues, and maintains homeostasis through essential mechanisms (64). The mucosa separates the intestinal lumen and deeper tissues, which are physically in touch with the gut lumen and comprise a single layer of polarized epithelial cells (65). Tight junctions between epithelial cells control paracellular permeability, preventing harmful substances from entering underlying tissues (66). Goblet cells secrete mucus that forms a protective layer, blocking pathogens and aiding their removal (67). Paneth cells release antimicrobial peptides (AMPs), controlling microbial populations (68). Dendritic cells and macrophages in the lamina propria produce TGF-β and IL-10, fostering Treg-mediated immune homeostasis, as shown in **Figure 3** (69). This harmonious system breaks down in IBD, much like a besieged fortress. Dysregulated immunity releases TNF-α and IL-17, which disrupt tight junctions, increase permeability, and permit translocation of microbes (70). Recent organoid-based epithelial injury models confirm that exposure to inflammatory cytokines reduces ZO-1, occludin, and claudin-1 localization, directly demonstrating junctional rearrangement observed in patient tissue (71, 72). In CD, Paneth cell defects, connected to NOD2 mutations, impair AMP production and exacerbate microbial dysbiosis (73). In UC, goblet cell dysfunction thins the mucus layer, exposing the epithelium to pathogens (73). SCFAs are decreased by microbial shifts, in turn impairing epithelial repair and Treg function (74). Microbial shifts modulate bile acid metabolism, altering FXR-dependent epithelial signals and disrupting barrier renewal (28). The barrier defects of CD are exacerbated by dysregulated bile acid metabolism, a novel disruptor that modifies microbial signalling (75). Antibiotics used in early life further disrupt microbiota, which leads to barrier failure (76).

### 2.3 Signaling pathways in inflammation

**Figure 4:**
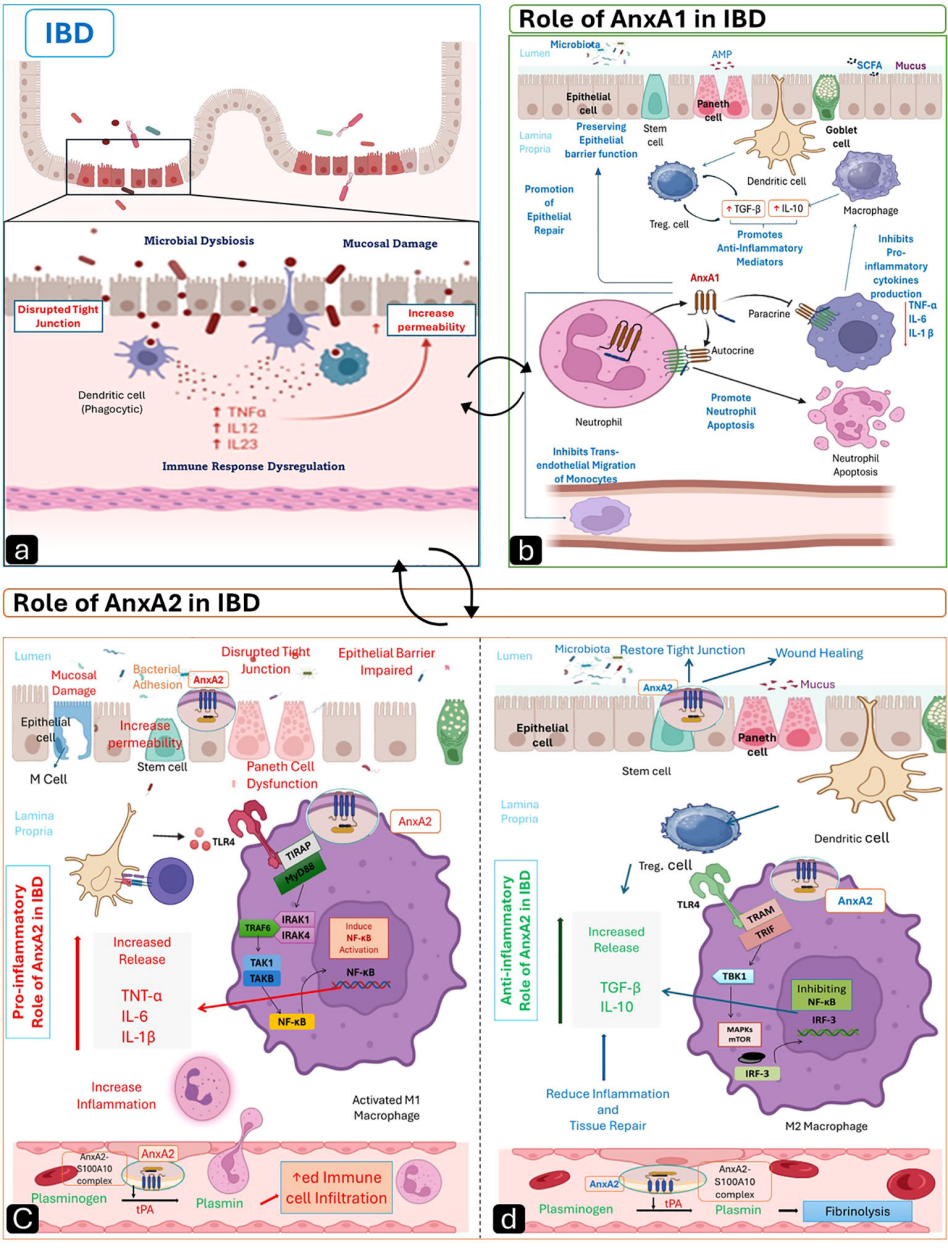
The role of AnxA1 and AnxA2 in IBD. **(A)** Intestinal mucosal alteration and immune response dysregulation in IBD. **(B)** Role of AnxA1 in IBD: AnxA1 plays an anti-inflammatory role by interacting with FPR2 on neutrophils and macrophages, thereby inhibiting pro-inflammatory cytokines, and promoting anti-inflammatory cytokines (TGF-β, IL-10), while also inducing neutrophil apoptosis. It maintains epithelial barrier integrity by stabilizing tight junctions and supports epithelial repair through wound healing and reduced monocyte migration. **(C)** Pro-inflammatory role of AnxA2: AnxA2 interacts with S100A10, activating NF-κB, which in turn increases the production of pro-inflammatory cytokines (TNF-α, IL-6, IL-1β) and promotes plasminogen activation, ultimately leading to immune infiltration, tight junction disruption, and increased epithelial permeability. **(D)** Anti-inflammatory role of AnxA2: AnxA2 inhibits NF-κB, promotes IL-10 and TGF-β, enhances fibrinolysis, and aids tight junction restoration and tissue repair. Created by the author using BioRender and Microsoft PowerPoint.

Intracellular signaling pathways orchestrate the activation, amplification, and chronicity of intestinal inflammation, as illustrated in **Figure 4A**. NF-κB activation, triggered by pro-inflammatory cytokines such as TNF-α, IL-6, and IL-1β, stimulates the transcription of pro-inflammatory genes, including cyclooxygenase-2 (COX-2), IL-6, and IL-8. These genes, in turn, promote neutrophil infiltration and epithelial damage (77). Mitogen-activated protein kinases (MAPKs), like p38, JNK, and ERK, stimulate Th17 differentiation and neutrophil ROS production through mediating cytokine signalling (51). IL-6 and IL-23 activate the Janus kinase-signal transducer and activator of the transcription (JAK-STAT) signaling pathway, which sustains immune cell survival and cytokine persistence, particularly in CD (78, 79). Multi-omics analyses show that JAK-STAT upregulation co-occurs with metabolic rewiring of inflammatory monocytes in UC, reinforcing cytokine persistence (80). Chronicity is sustained through a feedback loop created by MAPK-driven NF-κB activation or JAK-STAT synergy with IL-23 signaling. While smoking may increase NF-κB in CD, IL23R variants improve Th17 responses (81, 82). Single-cell datasets reveal spatial clustering of NF-κB-high myeloid cells near damaged epithelial zones, clarifying why anti-TNF therapies fail when these niches dominate (47).

## 3 Structure and function of AnxA1 and AnxA2

AnxA1 and AnxA2 are calcium-dependent phospholipid-binding proteins belonging to the annexin superfamily. Currently, 13 common vertebrate annexins have been identified (Annexin A1–A13). AnxA1 and AnxA2 are the first human annexins to be cloned (83, 84).

### 3.1 Structural characteristics of AnxA1 and AnxA2

AnxA1 is a 37-kDa phospholipid-binding protein dependent on calcium (85). Formerly referred to as lipocortin-1, renocortin, macrocortin, and lipomodulinb (84). AnxA2 is a 36-kDa phospholipid-binding protein dependent on calcium (19, 86). Formerly referred to as calpactin I, lipocortin II, placental anticoagulant IV, chromobindin VIII, and p36 (87). AnxA1 is a monomeric, amphipathic cytoplasmic protein comprising 346 amino acids. In comparison, AnxA2 is a monomeric protein containing 339 amino acids that binds to calcium and phospholipids (88). Both proteins share a conserved structural organization of two main domains: a protein core domain of the COOH-terminal (C-terminal) that mediates Calcium-dependent phospholipid binding and typically contains four homologous repeats, each consisting of five α-helices, and a flexible NH2-terminal (N-terminal) that confers regulatory functions relevant to inflammation (89, 90).

The length and sequence of the N-terminal domains of AnxA1 and AnxA2 vary and have short tails of 33 and 42 residues, respectively (90). They contain specific binding sites and phosphorylation sites for S100 proteins (S100A11 for AnxA1 and S100A10 for AnxA2). In AnxA1, the N-terminal region interacts with the S100 protein-ligand S100A11 (91). In contrast, AnxA2 interacts with S100A10, forming a heterotetramer that enhances membrane interaction and supports fibrinolysis (92). The N-terminal domain in AnxA1 contains phosphorylation sites (Ser/Thr) and possesses several proteolytic motifs, and a truncated N-terminal fragment is often found in inflammatory fluids (93).In AnxA2, phosphorylation at Tyr23, Ser25, and Ser11 modulates membrane association, nuclear shuttling, and extracellular translocation, functions that are increasingly recognized as context-dependent regulators of inflammation (94).

### 3.2 Distribution and regulation of AnxA1and AnxA2

#### 3.2.1 AnxA1 distribution and regulation

AnxA1 is distributed and expressed across various organs and tissues, mainly in epithelial and endothelial cells. It is present in all leukocytes except B lymphocytes, with the highest levels in monocytes and neutrophils. AnxA1 is found in plasma, seminal fluid, and intracellular and extracellular locations (95), and can be released into the bloodstream, acting in autocrine and paracrine signalling (96). AnxA1 secretion occurs via non-conventional mechanisms involving ATP-binding cassette (ABC transporter) transport, phosphorylation at serine-27 in Pituitary cells, and gelatinase granules in neutrophils (97). AnxA1 displays an atypical subcellular distribution, primarily found in the cytoplasm, with a smaller but functionally significant fraction found in both the inner and outer plasma membrane leaflets (98, 99).

In its resting state, the AnxA1 N-terminal region is concealed, and its release requires extracellular calcium stimulation (~1 mM), enabling AnxA1 to translocate to the outer leaflet of the plasma membrane, and engage in autocrine and paracrine signaling (17, 100). Recent studies show that membrane-exposed AnxA1 interacts directly with FPR2/ALX to induce efferocytosis, macrophage IL-10 production, and resolution-phase reprogramming (101).

AnxA1 is induced and regulated by glucocorticoids (GCs) (102), AnxA1 acts through the formyl peptide receptor type 2/lipoxin A4 receptor (FPR2/ALX). FPR2/ALX, along with FPR1 and FPR3, belongs to a family of G-protein-coupled receptors with significant sequence homology (20, 103). During inflammatory disease, GCs regulate AnxA1 levels. In diseases characterized by high cortisol levels, such as Cushing's disease, AnxA1 levels are upregulated. Meanwhile, conditions with low cortisol levels, such as Addison's disease, are associated with reduced AnxA1 levels compared to healthy controls (104).

#### 3.2.2 AnxA2 distribution and regulation

AnxA2 is produced by endothelial, trophoblast, epithelial, and tumor cells, macrophages, monocytes, and dendritic cells (105). AnxA2 has diverse subcellular localizations and can be present in the cell membrane, cytoplasm, or nucleus (106, 107). The S100A10–AnxA2 heterotetramer, formed by two AnxA2 subunits bound to an S100A10 dimer via the first 12 N-terminal amino acids of AnxA2. This heterotetramer is localized to the cell membranes (92, 105). AnxA2 is regulated by post-translational modifications and subcellular localization, influencing extracellular translocation and cellular roles (106); (i) Tyr23 phosphorylation drives extracellular translocation and NF-κB–linked inflammatory signaling (108). (ii) Serine acetylation stabilizes tetramer formation and supports fibrinolytic activity (92, 109).

Recent single-cell and spatial studies have highlight strong enrichment of AnxA2 in epithelial and macrophage populations in active UC, where extracellular AnxA2 correlates with pro-inflammatory cytokine programs (25, 110).

### 3.3 Functional roles of AnxA1

AnxA1 exerts potent anti-inflammatory and pro-resolving actions. It inhibits phospholipase A2 (PLA2) activity, reducing prostaglandin synthesis and macrophage activation, decreasing the acute inflammatory response (111). AnxA1 actions are mediated largely through FPR2/ALX and downstream signaling pathways that regulate innate and adaptive immune responses (112).

In neutrophils, AnxA1 inhibits activation and reduces arachidonate release, enzyme release, and adhesion to endothelial cells (113). It induces transient calcium fluxes and L-selectin shedding, reducing cell transmigration and promoting neutrophil apoptosis, which is vital for controlling inflammation (97, 114). In monocytes and macrophages, exogenous AnxA1 downregulates monocyte accumulation in inflammatory responses, inhibits phagocytic activity, adhesion, and trans-endothelial migration of monocytic cells, and promotes apoptosis (115–117). AnxA1 inhibits nitric oxide production and inducible nitric oxide synthase expression, which leads to an increase in IL-10 protein and reduces IL-12 levels (118). In lymphocytes, AnxA1 has an anti-proliferative effect on T-lymphocytes but not B-lymphocytes, as AnxA1 does not express in B-lymphocytes (111, 119). It suppresses mitogen-stimulated T-cell proliferation and inhibits antigen presentation, interfering with the activation of antigen-specific T-lymphocytes (120–123). Furthermore, AnxA1 is expressed and functions in several inflammation-related cell types, including endothelial cells, epithelial cells, mast cells, and synoviocytes (111). Recent evidence shows that AnxA1 also drives macrophage polarization toward pro-resolving M2 phenotypes via STAT3/IL-10 pathways, enhancing tissue repair and epithelial restitution (80, 101). Single-cell profiling in colitis further confirms enrichment of ANXA1 in epithelial and CD4^+^ T-cell subsets involved in mucosal healing (124).

AnxA1 is expressed in endothelial cells and localized to the nucleus; however, its mobilization is not evident during short-term neutrophil adhesion assays (125). In epithelial cells like A549, AnxA1 regulates cell proliferation (126). It mediates the anti-proliferative actions of GCs by inhibiting prostaglandin release and affecting cytosolic phospholipase A2 activation through epidermal growth factor signalling (127, 128). In Mast cells, AnxA1 is found in perivascular tissues, express AnxA1 is expressed, though at lower levels. GCs modulate increased AnxA1 expression in mast cells during inflammation (129). Significantly, AnxA1 in mast cells can inhibit histamine release, contributing to the stabilization effects of interleukin-2 (130). Given the mast cell's role in initiating and potentially resolving inflammation, AnxA1's function in these cells is expected to be significant and warrants further investigation (131).

### 3.4 Functional roles of AnxA2

AnxA2 is involved in membrane trafficking and organization, participating in processes such as exocytosis, endocytosis, and the formation of tight junctions (105, 132). AnxA2 binds with F-actin and plays a role in actin dynamics, organization, and membrane cytoskeletal rearrangement (92, 106). In Fibrinolysis regulation, AnxA2 heterotetramer binds plasminogen and tPA on the cell surface, promoting plasmin generation and regulating fibrinolysis (89, 108). AnxA2 has a well-established dual role in inflammation:

#### 3.4.1 Pro-inflammatory context (extracellular AnxA2)

The externalization of AnxA2/A2t activates the NF-κB signaling pathway, leading to the production of pro-inflammatory cytokines, including TNF-α, IL-6, and IL-1β. This transition is regulated by Tyr23 phosphorylation, which serves as a molecular switch driving its translocation and pro-inflammatory signaling (94). Strongly enriched in epithelial cells during active UC (25, 110). Organoid injury models show that AnxA2 upregulation coincides with tight-junction disruption and epithelial stress responses (133).

#### 3.4.2 Anti-inflammatory/repair context (intracellular AnxA2)

Intracellular AnxA2 performs the opposite function, supporting barrier repair by stabilizing ZO-1, occludin, and claudin-1. It also facilitates fibrinolysis and wound resolution through plasmin generation. Recent organoid studies have further demonstrated that AnxA2 enhances β1-integrin recycling, thereby promoting epithelial cell migration and tissue repair (47).Whether AnxA2 is protective or inflammatory depends on its subcellular localization, phosphorylation state, and interaction with innate immune pathways, including TRAM–TRIF versus MyD88 signaling biases in macrophages (94).

## 4 Roles of AnxA1 and A2 in IBD pathogenesis

AnxA1 and AnxA2 are key endogenous regulators of inflammation signaling, exhibiting dysregulated expression that contributes to the chronic intestinal inflammation observed in IBD pathogenesis (84). Both proteins influence epithelial integrity, macrophage programming, neutrophil trafficking, and cytokine amplification loops that are relevant to UC and CD. Their context-dependent actions in inflammation, immunity, and tissue repair position them at the intersection of molecular pathways central to IBD pathogenesis, as supported by classical and modern mechanistic studies (134, 135). A comparative overview of AnxA1 and AnxA2 expression patterns and functional roles is summarized in **Table 1**.

**Table 1:** Comparative patterns of AnxA1 and AnxA2 across IBD stages & models.

Disease stage/model	AnxA1 expression & functional pattern	AnxA2 expression & functional pattern
Active ulcerative colitis (human mucosa, biopsies)	Frequently elevated in epithelium and lamina propria in several transcriptomic/proteomic studies; linked to epithelial signals and pro-resolution pathways (FPR2/ALX/IL-10) in some cohorts (24, 53, 94).	Upregulated in UC mucosa in proteomic/transcriptomic datasets; A2t externalization / S100A10 interactions are associated with NF-κB activation and higher local cytokines in UC (25, 94).
Ulcerative colitis remission/healing (human)	Partial restoration of AnxA1 expression, when measured, was associated with improved clinical/endoscopic scores in small cohorts (24, 133).	Often reduced vs active disease, though some samples retain elevated A2 in areas prone to relapses (limited human longitudinal data) (24, 25).
Active Crohn’s disease (human mucosa, biopsies)	Mixed reports: several studies identify increased ANXA1 transcripts in CD lesion panels and as part of diagnostic gene sets; functional work suggests defective AnxA1 secretion (non-conventional/ABC transporter) in CD that impairs FPR2 signaling (94, 124).	Variable — moderate expression in inflamed segments in some datasets; may show reparative roles in fistula/repair contexts (mechanistic, limited human validation) (72, 94).
Crohn’s remission/healing (human)	Reports of restored AnxA1 activity in limited samples linked to repair; human longitudinal data are limited (72, 124).	AnxA2 may participate in epithelial restitution via integrin trafficking in repair models; human confirmation is scarce (72).
DSS colitis (mouse, acute chemical model)	Protective role: AnxA1 deficiency worsens colitis; Ac2–26 or AnxA1 nanoparticles improve histology, ↑IL-10 and efferocytosis, ↓inflammation (94, 101, 133).	Often upregulated in inflamed tissue; can contribute to both inflammation (NF-κB axis) and repair depending on cell type and timing (94, 136).
TNBS / Th1-skewed colitis models	AnxA1/Ac2–26 reduces Th1/Th17 inflammation and mucosal damage in several preclinical studies (94, 101).	AnxA2 effects are model-dependent; reported roles include matrix remodeling and modulation of inflammation (94).
Intestinal organoids / epithelial-repair models (*in vitro* / organoid)	Promotes wound closure and tight-junction integrity (ZO-1/occludin/claudins) in inflamed epithelium models; AnxA1 influence tested in stem-cell epithelium assays (71, 72, 133).	Regulates β1-integrin internalization and epithelial migration — organoid and cell studies validate role for AnxA2 in epithelial resealing (71, 72).
Single-cell / spatial transcriptomics (scRNA, spatial) findings	Detected in epithelial compartments and immune subsets (e.g., some CD4^+^ T-cell populations, Tregs), depending on the dataset and disease context (26, 124).	Enriched in macrophage clusters and certain stromal/epithelial cells in UC atlases; ligand–receptor analyses show ANXA1–FPR interactions across fibroblast–macrophage axes (25, 26, 137).
Proteomics/serum / fecal biomarker studies	Included in multi-gene expression panels (e.g., 3-gene panel discriminating CD vs UC, where ANXA1 contributed to discrimination, AUC ~0.84 for the panel); limited studies report AnxA1 protein in plasma/biopsies (24, 53).	Detected by tissue proteomics as UC-associated; suggested as a candidate relapse predictor in some proteomic datasets, but no standardized clinical assay yet (25, 138).

### 4.1 AnxA1 and AnxA2 in inflammatory diseases

Inflammation is generally a protective immune response; however, when it becomes uncontrolled or untreatable, it can cause further damage and lead to chronic diseases, including asthma, lung injuries, multiple sclerosis, rheumatoid arthritis, rhinitis, chronic obstructive pulmonary disease, Crohn's disease, ulcerative colitis, prostatitis, endocrine disorders, psoriasis, vasculitis, and a range of autoimmune and degenerative disorders such as obesity, Alzheimer's disease, and atherosclerosis (84, 139). Annexins serve as endogenous inflammation-limiting mediators, counterbalancing pro-inflammatory signaling and helping to restore homeostasis (112). AnxA1 is well-established as a glucocorticoid-induced pro-resolving mediator (122, 140). It suppresses macrophage pro-inflammatory cytokine release, promotes IL-10 production, and inhibits PLA2 activity (141).

In Asthma, AnxA1 and SPMs levels are lower in wheezy newborns than in controls, with higher serum IgE levels and eosinophilia (142). AnxA1-derived peptides, such as Ac2-26, reduce CRTH2 expression, PGD2 levels, and eosinophil accumulation (143, 144). In respiratory illness, AnxA1 is decreased in lung tissue and bronchoalveolar lavage fluid (145), and proteolytic degradation during lung injury further decreases its availability (146).

In multiple sclerosis (MS), high-dose GCs and immunomodulatory therapies are the gold-standard approaches for managing acute relapses and promoting recovery (147) AnxA1 was elevated in treatment-naive relapsing/remitting MS (RRMS) patients, with a negative correlation to disease severity and progression, suggesting a compensatory inflammatory control mechanism (148). In rheumatoid arthritis (RA), AnxA1 is key in regulating inflammation, primarily through GC treatment. In treatment-naive RA patients, lower AnxA1 levels are associated with reduced Foxp3 expression and increased IL-17 production, underscoring the importance of AnxA1 in immune regulation and anti-inflammatory processes in RA pathogenesis (19, 99). In Alzheimer's disease (AD), AnxA1 offers protection against AD by degradation and clearance of β-amyloid (Aβ) and reducing neuroinflammation, making it a potential therapeutic option in AD (149).

AnxA2 is a multifaceted protein involved in various cellular processes, including inflammation. AnxA2 has both pro-inflammatory and anti-inflammatory effects depending on the context. Initially. It maintains vascular integrity in acute inflammation by interacting with Actin and VE-cadherins, which regulate vascular permeability (105), and repairs damaged cell membranes, regulates inflammasome activation, and controls inflammatory responses (132, 150). Moreover, AnxA2 helps recruit inflammatory cells like neutrophils and monocytes to injury sites by interacting with molecules of CD44 (86, 105).

In Rheumatoid Arthritis (RA), AnxA2 is significantly higher in plasma, synovial fluid, and synovial tissues, acting as an upstream regulator of cytokines and chemokines driving RA pathogenesis (19). Inhibiting AnxA2 can mimic glucocorticoid treatment effects, involving AnxA1 expression and reducing pro-inflammatory cytokines (19). In Alzheimer's disease (AD), AnxA2 has a dual role in anti-inflammatory and pro-inflammatory disease. In early stages, it protects internal membranes and limits vascular permeability, while in later stages, it may promote inflammation as a binding site for inflammatory ligands, which are also involved in the autophagosome's degradation of Aβ1–42 aggregates (151). In lupus nephritis (LN) and other renal inflammatory disorders, AnxA2 acts as an autoantigen that triggers the immune response and promotes tPA-induced NF-kB activation, leading to kidney inflammation and damage (86, 152). Anti-AnxA2 antibodies are present in patients with antiphospholipid syndrome (108, 153). In sepsis, AnxA2 plays diverse roles in inflammatory settings, influencing different pathways within the inflammasome system (105). It is also overexpressed in acute promyelocytic leukaemia (108). Additionally, AnxA2 can inhibit fibrinolysis by hindering plasmin-mediated fibrin polymer lysis and plasmin activity, contributing to thrombosis (92, 153).

### 4.2 AnxA1’s regulation in IBD pathogenesis

AnxA1 exerts anti-inflammatory and pro-resolving actions in IBD by modulating cytokine responses, preserving epithelial integrity, and promoting mucosal healing, as shown in **Figure 4B**. Both classical animal studies and modern high-throughput epithelial models support its relevance (133).

AnxA1 is secreted through a non-conventional pathway that involves ATP-binding cassette (ABC) transporters and gelatinase granules, which are stimulated by GCs signalling (154, 155). It interacts with the FPR2/ALX on immune and epithelial cells. Through this axis, AnxA1 suppresses the production of pro-inflammatory cytokines (TNF-α, IL-1β, and IL-6) and enhances the release of anti-inflammatory cytokines (IL-10 and TGF-β). Reduced AnxA1 expression in IBD correlates with barrier dysfunction, increased permeability, and disease severity (156, 157). Multi-omics studies further indicate that reduced AnxA1 expression in IECs is associated with impaired efferocytosis pathways and heightened NF-κB signatures in UC and CD (24, 80).

AnxA1 stabilizes tight junctions between IECs, thereby preserving epithelial barrier function. Reduced AnxA1 levels contribute to barrier dysfunction and increased intestinal permeability, characteristic of IBD (83, 133). AnxA1 supports epithelial restitution by guiding cell migration and wound closure, facilitating cytoskeletal rearrangements, and restoring tight junction proteins such as claudins and occludins. High-throughput organoid studies confirm that AnxA1 supplementation accelerates epithelial repair following cytokine-induced injury (133, 135).

### 4.3 AnxA2’s dual roles in IBD pathogenesis

AnxA2 exhibits both pro-inflammatory and anti-inflammatory roles in IBD, depending on cellular context and phosphorylation state. Understanding the duality of AnxA2 is essential because shifts between M1 and M2 macrophage states, along with their associated cytokine patterns define the inflammatory pathway in IBD, as illustrated in **Figure 4**.

#### 4.3.1 Pro-inflammatory mechanisms in IBD

AnxA2 promotes inflammation via many pathways, as illustrated in (**Figure 4C**), including:

− Activating NF-κB in activated M1 macrophages encourages the release of pro-inflammatory cytokines, TNF-α, IL-6, and IL-1β (86, 92).

− AnxA2 interacts with S100A10, forming an AnxA2-S100A10 complex, which, through tPA, facilitates the conversion of plasminogen to plasmin on the surface of endothelial cells. This process increases immune cell infiltration, leading to heightened inflammation and tissue damage (105, 158).

− Plasmin enhances immune cell infiltration, tight junction disruption, and epithelial permeability by activating pro-matrix metalloproteases (MMPs) (105, 107).

− AnxA2 phosphorylation at Tyr23 enhances membrane localization and amplifies inflammatory signaling, a mechanism supported by recent phospho-proteomic studies (159).

− In IBD mucosa, elevated AnxA2 expression correlates with neutrophil-rich inflammation and disrupted epithelial junctions (24).

#### 4.3.2 Anti-Inflammatory and reparative mechanisms in IBD

In contrast, AnxA2 also contributes to inflammation resolution, as illustrated in (**Figure 4D**), through:

- AnxA2 acts as an anti-inflammatory mediator by inhibiting NF-κB in M2 macrophages, which promotes anti-inflammatory cytokine production, enhances fibrinolysis, and aids tight junction restoration and tissue repair (105, 108).
- Activation of the TRAM-TRIF endosomal pathway by promoting the translocation of Toll-like receptor 4 (TLR4) into the endosomal membrane. Concurrently, it suppresses MyD88–NF-κB signaling, increasing the release of anti-inflammatory cytokines, particularly IL-10 and TGF-β.138,162.
- Enhancing fibrinolysis by facilitating plasmin generation, which is vital for fibrin degradation, angiogenesis, and regulating hemostasis (108).
- Regulation of β1-integrin internalization, enabling rapid epithelial migration and resealing of injury sites, thereby restoring endothelial tight junctions, maintaining mucosal integrity and permeability (107, 160).
- Single-cell analyses show that AnxA2 is enriched in epithelial clusters associated with restitution and wound-healing phenotypes in UC (47).

### 4.4 Annexin and microbiota interactions in IBD

Recent data indicate a bidirectional relationship between annexins (particularly AnxA1) and the intestinal microbiota that may influence IBD severity and recovery. Most directly, a preclinical study demonstrated that manipulating of AnxA1 levels alters gut microbial composition and modifies susceptibility to DSS-induced colitis, supporting a causal role for AnxA1 in microbiota-host crosstalk (136). Complementary multi-omics and metabolomics studies in human IBD cohorts identify reproducible alterations in fecal and serum metabolite profiles that track disease activity and could mechanistically link microbial metabolites to host resolution pathways (1, 27, 28,).Mucosal expression analyses that include ANXA1 further show disease-stage–specific patterns, indicating that annexin expression can be measured alongside microbiome signatures in patient biopsies (24, 124). Extracellular vesicles and other microbial–host signaling modalities have been proposed as routes by which microbiota and host proteins communicate during inflammation and repair, suggesting plausible mechanisms for annexin-mediated effects on microbial ecology and metabolite exchange (161). Collectively, these studies justify the explicit integration of annexin quantification with microbiome and metabolome profiling in future IBD cohorts to test whether annexin–microbiome axes predict relapse, treatment response, or mucosal healing. Recommended approaches include longitudinal sampling, paired mucosal and stool multi-omics, and gnotobiotic or fecal-transfer experiments to test causality (27, 28, 136).

## 5 Emerging diagnostic and therapeutic strategies targeting AnxA1 and A2 in IBD

The persistent diagnostic delays, limited biomarker specificity, and variable treatment responses continue to challenge the management of IBD (13). Recent advances in molecular profiling, proteomics, and single-cell technologies have renewed interest in AnxA1 and AnxA2 as subtype-specific biomarkers and therapeutic targets, offering precision-oriented and non-invasive approaches to improve early diagnosis and treatment personalization (84, 135).

### 5.1 Current challenges and advances in IBD diagnosis

IBD diagnosis is hindered by the reliance on invasive procedures and the lack of highly specific, laboratory-based tests. Colonoscopy remains the diagnostic gold standard, but it is costly, invasive, which limits its use as a routine screening method (13). Common non-invasive biomarkers like fecal calprotectin and C-reactive protein (CRP) cannot accurately reflect disease activity, distinguish CD from UC, or track mucosal inflammation (22). Recent Multi-omics studies demonstrate that annexin family proteins, particularly AnxA1 and AnxA2, provide disease-specific signatures that complement existing biomarkers and may improve subtype stratification pending validation in larger clinical cohorts (24).

CD mucosa exhibits significantly lower levels of AnxA1 expression due to impaired non-conventional secretion via ABC transporters, which reflects defective FPR2/ALX signaling and correlates strongly with Crohn's Disease Activity Index (CDAI) scores (5, 162). Low circulating and mucosal AnxA1 levels in plasma and biopsies by enzyme-linked immunosorbent assays (ELISA) and immunohistochemistry (83). In UC, AnxA1 expression fluctuates with disease stage, partially restored in remission and significantly decreased in active disease, correlating with Mayo Clinic scores (22).

AnxA2, conversely, shows robust overexpression in UC (163), where epithelial A2t externalization and NF-κB activation, as well as AnxA2 abundance, are confirmed by quantitative PCR and mass spectrometry-based proteomics (152, 164). This elevation correlates with mucosal TNF-α levels and endoscopic severity. In CD, AnxA2 expression is more moderate and enriched in resolving lesions, aligning with its context-dependent anti-inflammatory function (160). These disease-specific annexin expression patterns provide a mechanistic basis for their potential diagnostic utility, supporting the development of ELISA-based subtype differentiation approaches that require further clinical validation (62).

Recent quantitative evaluations further support their diagnostic potential. In biopsy-based analyses, AnxA1 immunostaining distinguished IBD from controls with an AUC of 0.82, sensitivity of 78%, and specificity of 74% (130 ).Meanwhile, single-cell and proteomic classifiers integrating AnxA1 improved performance to an AUC of ~0.90 in multi-gene panels (24). For AnxA2, UC-specific overexpression yielded an AUC of 0.86 for differentiating active UC from remission (94), and plasma, and tissue correlation studies reported sensitivities and specificities of 72% and 70%, respectively (72). Compared with current clinical markers, fecal calprotectin (AUC 0.85–0.90; sensitivity 80–95%; specificity 70–82%) and CRP (sensitivity 50–60%), annexin-based assays provide complementary, subtype-oriented precision rather than replacement, supporting their emerging role as adjunctive biomarkers in IBD evaluation (27, 28).

### 5.2 AnxA1-targeted therapeutic strategies

AnxA1’s anti-inflammatory and pro-resolving properties position it as a compelling therapeutic target. As shown in **Table 2**, the molecular actions of AnxA1 differ between CD and UC, reflecting distinct defects in efferocytosis, cytokine resolution, and epithelial repair. In CD, impaired FPR2/ALX signaling caused by reduced AnxA1 secretion results in persistent neutrophil infiltration and inadequate resolution (5). AnxA1-derived peptides such as Ac2–26 restore resolution pathways by enhancing IL-10 production, improving efferocytosis, and reducing inflammatory cytokine release (9, 168).

**Table 2:** Mechanism-based and evidence-verified therapeutic strategies targeting AnxA1/A2 in IBD.

Treatment type / category	Intervention	Mechanism of action	Study evidence (design; species)	Effect direction (↑/↓)	References
Mucosal Repair / Targeted Delivery	Ac2–26 mesoporous microparticles (Eudragit–SBA-15-Ac2-26)	Annexin A1 mimetic; FPR1/2 activation → ↑ efferocytosis, ↑ IL-10, promotes epithelial repair (tight-junction re-organization)	Preclinical experimental; mouse DSS colitis	↓ DAI, ↓ TNF-α/CXCL1; ↑ IL-10, ↑ colon length, improved histology	(133)
Resolution Promotion / Myeloid Tuning	Small-molecule FPR2/ALX modulators (oral)	FPR2/ALX modulation (biased agonism) → enhanced efferocytosis and myeloid reprogramming	Preclinical animal study; mouse models	↓ DAI and histologic scores; restored cytokine balance	(138)
Resolution Promotion / Efferocytosis Enhancement	Columbamine-like biased FPR2 agents	Biased FPR2 signaling (non-canonical ligands) → ↑ macrophage efferocytosis → ↓ intestinal inflammation	Preclinical *in vitro* + mouse models	↑ Efferocytosis; ↓ colitis severity	(109)
Targeted Anti-Inflammatory Delivery	Polyphenol-assisted IL-10 mRNA nanoparticles	Local mRNA / nanoparticle delivery → colonic IL-10 expression → mucosal healing	Preclinical nanodelivery study; mouse models	↓ Inflammation; improved histology	(165)
Annexin–Microbiota Interplay (Mechanistic)	AnxA1 overexpression / knockout models	Altered AnxA1 levels modulate microbiota composition and colitis susceptibility	Preclinical DSS models with 16S rRNA sequencing	AnxA1 ↑ → protective; AnxA1 ↓ → susceptible microbiota shift	(136)
Clinical Comparator — Biologic (Context)	Ustekinumab	IL-12/23 blockade → immune modulation; benchmarks AnxA strategies	Clinical retrospective multicenter study; n = 50 patients (Parra 2024)	Clinical remission and endoscopic improvement	(166)
Clinical Comparator — JAK Inhibitor (Context)	Tofacitinib	JAK pathway inhibition → anti-inflammatory / anti-fibrotic maintenance	Clinical observational multicenter cohort; n = 111 patients (Macaluso 2024)	Clinical response and maintenance; safety profile described	(167)

Ac2–26 mitigates active inflammation in ulcerative colitis by lowering TNF-α and IL-1β, while nanoparticle-based formulations improve stability, colon-selective delivery, and therapeutic efficacy (169). Direct therapeutic approaches include AnxA1 mimetics (Ac2-26), whereas indirect approaches involve lipoxin A4 analogues and FPR2/ALX agonists that amplify endogenous AnxA1 signalling (84). Preclinical studies demonstrate that indirect agonists attenuate IL-17–mediated injury and mucosal damage in TNBS colitis. Delivery systems include systemic, rectal, and nanoparticle-based routes, each of which improves tissue targeting and stability (135). Early human studies suggest that higher mucosal AnxA1 during UC remission predicts improved anti-TNF response, highlighting its translational relevance (21).

### 5.3 AnxA2-targeted therapeutic strategies

The dual and context-dependent roles of AnxA2 necessitate therapeutic strategies tailored to the disease phenotype. In UC, where A2t externalization and NF-κB activation drive early epithelial injury, TNF-α/IL-6 release, and tight junction disruption, AnxA2 represents a pro-inflammatory mediator (105, 163). Anti-A2t antibodies reduce NF-κB-dependent cytokine storms and improve clinical and histological indices in DSS colitis (152). Small-molecule inhibitors that disrupt AnxA2–S100A10 interactions limit integrin-β1 signaling, reducing immune infiltration and epithelial damage (160).

In CD, AnxA2 exhibits an anti-inflammatory profile through PI3K/AKT activation, tight junction stabilization, and the promotion of IL-10 and TGF-β (92, 170). PI3K/AKT agonists and IL-10–inducing nanoparticle systems enhance AnxA2-mediated repair pathways, especially in fistulizing or fibrotic CD phenotypes (see **Table 2**) (108). Although human studies remain limited, the consistent association between mucosal AnxA2 overexpression and UC relapse risk suggests that AnxA2 inhibitors may serve as adjuncts to biologics in selected patients (163).

### 5.4 Overcoming challenges in IBD management

Integrating AnxA1 and AnxA2-based diagnostics and therapies into IBD management may help overcome persistent clinical challenges. Dependence on Colonoscopy and non-specific biomarkers delays accurate diagnosis and increases healthcare burden (171). Subtype-specific profiles of AnxA1 and A2 (low AnxA1 in CD, high AnxA2 in UC) suggest a possible role in future precision, non-invasive diagnostics using proteomics or ELISA platforms once validated through standardized clinical assays, offering improved specificity compared with CRP (22). However, standardization of annexin assays and cross-cohort validation remain essential before clinical adoption, and AI-driven multi-omics approaches may accelerate this process (62).

Due to side effects and limited resolution promotion, up to 40% of IBD patients exhibit primary non-response to biologics, driven by unresolved inflammation, dysbiosis, and immune heterogeneity (163). **Table 2** demonstrates how AnxA1-targeted treatments (efferocytosis enhancement, IL-10 induction) and AnxA2-modulating therapies (inflammation suppression in UC, tissue repair in CD) may complement existing biologics such as anti-TNF agents, JAK inhibitors, and anti-integrins (22, 172). Delivery barriers, especially colon-specific targeting, remain an obstacle for peptide and nucleic-acid-based therapies, necessitating nanoparticle optimization (160). Future directions include integrating annexin-guided biomarker strategies with precision medicine frameworks to refine patient selection and improve therapeutic durability (21).

Safety considerations are critical for annexin-directed strategies. Annexin A2 (AnxA2) functions as a plasminogen/tPA co-receptor, thereby influencing fibrinolysis and vascular homeostasis. Perturbing this axis could alter the risk of thrombosis or bleeding (20, 173). AnxA2 has been implicated in tumor invasion and metastasis in several cancers, so chronic or systemic activation of AnxA2 pathways requires caution in populations at risk for malignancy (81, 137). Post-translational modification (e.g., Tyr23 phosphorylation) modulates AnxA2 signaling and can enhance downstream proliferation/invasion programs in epithelial cells, indicating that phosphorylation-dependent effects may produce off-target consequences (174). Finally, AnxA2 participates in host–pathogen interactions and innate defense, so broad inhibition or augmentation might affect susceptibility to infection (20, 86). For these reasons, annexin-targeted therapeutics will require careful dose–response and safety profiling, tissue-targeted delivery (to limit systemic exposure), and preclinical evaluation of thrombotic, oncologic, and infectious risks before clinical trials.

## 6 Conclusion and future recommendations

### 6.1 Conclusion

IBD is characterized by a chronic, relapsing inflammatory disorder and complex pathogenesis that is driven by dysregulated immune responses, microbial imbalance, and epithelial barrier failure and continues to be a global health burden. The crucial contributions of AnxA1 and AnxA2 to the pathophysiology of IBD have been highlighted by this review. AnxA1 predominantly exerts anti-inflammatory and pro-resolving context through FPR2/ALX signaling, reducing excessive immune responses and preserving epithelial integrity. In contrast, AnxA2 displays context-dependent dual functions, regulates immune response and fibrinolysis under homeostatic or resolving conditions while enhancing NF-κB activity, plasmin generation, and barrier disruption in inflammatory milieus. Together, these proteins shape macrophage polarization, epithelial restitution, and cytokine signaling networks that are dysregulated in IBD.

AnxA1 and AnxA2 also show potential as non-invasive diagnostic biomarkers, offering subtype-specific insights that complement, rather than replace existing tools such as CRP, fecal calprotectin, and endoscopic/colonoscopic score. However, current evidence remains preliminary, as most findings derive from small cohorts, single-cell datasets, or preclinical models. Assay variability, lack of standardized thresholds, and differences in sample handling continue to limit clinical translation. Therefore, the implementation of diagnostics will require harmonized protocols, cross-platform validation, and comparison against established markers.

Therapeutically, modulating AnxA1 and AnxA2 signaling presents a promising strategy to enhance mucosal healing and immune resolution. AnxA1-based mimetics (e.g., Ac2-26), FPR2/ALX agonists, and targeted nanoparticle formulations have shown anti-inflammatory benefits in experimental colitis. AnxA2-directed strategies, including the inhibition of A2t externalization in UC and the enhancement of fibrinolytic or IL-10 pathways in CD, provide mechanistically grounded avenues for intervention. Nonetheless, challenges remain, including peptide stability, delivery to inflamed colonic tissue, and the absence of validated clinical tests to detect them. Translation into practice will require integrated pharmacokinetic, safety, and dose-response studies across diverse patient populations.

### 6.2 Gaps, contradictions, heterogeneity

Despite growing interest, major knowledge gaps persist. Current reports occasionally show contradictory findings, such as variable AnxA1 expression across disease stages and inconsistent associations between AnxA2 levels and clinical activity. Population heterogeneity, including age, diet, microbiome composition, ethnicity, prior infection, smoking, and medication history, may account for these discrepancies and must be controlled in future work. Furthermore, the interactions between AnxA1/AnxA2 and cytokine networks (e.g., IL-23/Th17, TNF, and IL-10 pathways) remain insufficiently defined, limiting mechanistic inference.

### 6.3 Cohort study recommendations

To address these limitations, future research should prioritize well-designed prospective cohort studies of AnxA1/AnxA2-based biomarkers. Recommended design elements include:

- Standardized sampling protocols (blood, stool, biopsy) with pre-analytical controls.
- Parallel measurement of CRP, fecal calprotectin, and endoscopic indices to allow benchmarking.
- Stratification by key modifiers (sex, age, diet, smoking, microbiome features, biologic exposure).
- Longitudinal sampling to evaluate intra-individual stability and relapse prediction.
- Integration with multi-omics datasets to link protein levels with cell-type-specific expression and receptor signaling states.
- Validation across independent cohorts, including treatment-naïve, biologic-exposed, and pediatric groups.
